# Dual Energy Method for Breast Imaging: A Simulation Study

**DOI:** 10.1155/2015/574238

**Published:** 2015-07-13

**Authors:** V. Koukou, N. Martini, C. Michail, P. Sotiropoulou, C. Fountzoula, N. Kalyvas, I. Kandarakis, G. Nikiforidis, G. Fountos

**Affiliations:** ^1^Department of Medical Physics, Faculty of Medicine, University of Patras, 265 00 Patras, Greece; ^2^Radiation Physics, Materials Technology and Biomedical Imaging Laboratory, Department of Biomedical Engineering, Technological Educational Institute of Athens, Egaleo, 122 10 Athens, Greece; ^3^Department of Medical Laboratories, Technological Educational Institute of Athens, Egaleo, 122 10 Athens, Greece

## Abstract

Dual energy methods can suppress the contrast between adipose and glandular tissues in the breast and therefore enhance the visibility of calcifications. In this study, a dual energy method based on analytical modeling was developed for the detection of minimum microcalcification thickness. To this aim, a modified radiographic X-ray unit was considered, in order to overcome the limited kVp range of mammographic units used in previous DE studies, combined with a high resolution CMOS sensor (pixel size of 22.5 *μ*m) for improved resolution. Various filter materials were examined based on their K-absorption edge. Hydroxyapatite (HAp) was used to simulate microcalcifications. The contrast to noise ratio (CNR_*tc*_) of the subtracted images was calculated for both monoenergetic and polyenergetic X-ray beams. The optimum monoenergetic pair was 23/58 keV for the low and high energy, respectively, resulting in a minimum detectable microcalcification thickness of 100 *μ*m. In the polyenergetic X-ray study, the optimal spectral combination was 40/70 kVp filtered with 100 *μ*m cadmium and 1000 *μ*m copper, respectively. In this case, the minimum detectable microcalcification thickness was 150 *μ*m. The proposed dual energy method provides improved microcalcification detectability in breast imaging with mean glandular dose values within acceptable levels.

## 1. Introduction

Breast cancer is one of the most common causes of cancer death in the United States [1]. Diagnosis of this type of cancer in its early stage makes the treatment more effective [2–4]. Several imaging modalities, such as mammography, ultrasound, and digital tomosynthesis, have been used in diagnosing breast cancer. X-ray mammography is the gold standard method for early detection of breast carcinomas [5–8]. One of the principal indicators of breast cancer is microcalcifications (*μ*Cs). Although they are small in size (0.1–1 mm), their X-ray attenuation is higher than the surrounding breast tissue, making them visible. However, overlapping tissue structures make them very difficult to detect [9]. Dual energy (DE) X-ray imaging can suppress the contrast between adipose and glandular tissues improving the detectability of microcalcificat ions (*μ*Cs) [10, 11]. With this technique, images that are acquired with two different X-ray spectra are subtracted, resulting in an image with enhanced microcalcification information [12].

Several dual energy techniques have been evaluated for mammography [13, 14]. These studies showed that the minimum calcification thickness was 250 *μ*m or higher when mammographic X-ray units were used [13, 14]. Previous simulation study has shown that improved calcification detectability can be achieved by using higher energies than those in the mammographic range [10]. Furthermore, detectors such as charged coupled detectors (CCD) and complementary metal oxide semiconductor (CMOS) active pixel sensors (APS), with an effective pixel size smaller than that of current flat panel imagers (70–100 *μ*m), can improve the detection and characterization of microcalcifications [15–17].

To this aim, a dual energy method for the detection of the minimum microcalcification thickness was investigated, incorporating a modified radiographic X-ray unit, in order to overcome the limited kVp range of mammographic units, combined with a high resolution CMOS sensor (pixel size of 22.5 *μ*m) for improved resolution. The pair of energies providing the minimum detectable microcalcification thickness was determined through monoenergetic analytical modeling. Afterwards, the optimum irradiation conditions (X-ray tube high voltage, filter material according to their K-edge and thickness) were determined for polyenergetic spectra. The calcification contrast to noise ratio of the subtracted images (CNR_*tc*_) was calculated for various skin entrance doses. The DE method modeled in this study provides improved calcification detectability in breast imaging.

## 2. Materials and Methods

In order to determine the minimum detectable calcification thickness, a previously presented theoretical framework [13] was used for the numerical calculation of contrast to noise ratio corresponding to the subtracted calcification image. Simulation studies were developed based on monoenergetic and polyenergetic X-ray beams. Various parameters were examined, that is, X-ray filtration, entrance surface, and mean glandular doses. The methods for these computations are described in the following sections.

### 2.1. Monoenergetic X-Ray Beams

An investigation based on simulation studies was employed in order to determine the optimal pair of energies, that is, the low and high X-ray energy, required for the DE technique. Initially monochromatic X-ray beams were considered with energies in the range between 15 and 90 keV, at 1 keV increments. Within this investigation, an analytical model was developed in order to determine the CNR_*tc*_ corresponding to a calcification (*C*). In this model, the pair of energies maximizing the CNR_*tc*_ were estimated for calcifications of various thicknesses under the following assumptions: (i) X-ray photons follow a Poisson distribution [13] and (ii) the CNR_*tc*_ threshold value for detection of a calcification is equal to 3 [13].

In the present investigation, it was considered that the breast consists of adipose tissue of thickness *t*_*a*_, glandular tissue of thickness *t*_*g*_, and a cubic calcification of thickness *t*_*c*_. Under this consideration, the signals in the low- and high-energy images, *S*_*l*_ and *S*_*h*_, were expressed as follows [13]:

(1) SjEi=Rj·d2·ΦjEi·e−μ/ρaEiρata−μ/ρgEiρgtg−μ/ρcEiρctc·QDEEi·As,j=l,h,

where *R*_*j*_ is the unattenuated low-/high-energy X-ray exposure at the detector input (mR), *d* is the pixel size in centimeters (mm), Φ_*j*_(*E*_*i*_) is the unattenuated low- and high-energy photon fluence per unit of energy and exposure (photons/mm^2 ^keV mR) at the detector input, QDE(*E*_*i*_) is the quantum detection efficiency of the phosphor screen as a function of photon energy *E*, and *A*_*s*_ is the spectral compatibility between the optical spectrum of the phosphor and the sensitivity of the optical photon sensor. The energy-dependent mass attenuation coefficients for adipose tissue, glandular tissue, and calcifications are given by *μ*/*ρ*_*a*_(*E*_*i*_), *μ*/*ρ*_*g*_(*E*_*i*_), and *μ*/*ρ*_*c*_(*E*_*i*_), respectively.

Then, the signal-to-noise ratio in the low- and high-energy image signals SNR_*S*_*j*__ can be calculated by [13]

(2) SNRSj=Rjd2ΦjEie−μ/ρaEiρaT−ΔMgEitg−ΔMcEitcQDEEiAsRjd2ΦjEie−μ/ρaEiρaT−ΔMgEitg−ΔMcEitcQDEEiAs2,j=l,h,

where Δ*M*_*g*_(*E*_*i*_) = ((*μ*/*ρ*)*g*(*E*_*i*_)*ρ*_*g*_ − (*μ*/*ρ*)*a*(*E*_*i*_)*ρ*_*a*_) and Δ*M*_*c*_(*E*_*i*_) = ((*μ*/*ρ*)*c*(*E*_*i*_)*ρ*_*c*_ − (*μ*/*ρ*)*a*(*E*_*i*_)*ρ*_*a*_).

The noise variance in the subtracted calcification image signal is given by [13]

(3) $$\begin{array}{c} \sigma_{tc}^{2}=\sqrt{\frac{k_{cl}^{2}}{{SNR}_{S_{l}}^{2}}+\frac{k_{ch}^{2}}{{SNR}_{S_{h}}^{2}}}, \end{array}$$

where

(4) kcl=−ΔMgEhΔMgElΔMcEh−ΔMgEhΔMcEl,kch=ΔMgElΔMgElΔMcEh−ΔMgEhΔMcEl.

Therefore, the SNR of the subtracted calcification signal can be expressed as follows [13]:

(5) $$\begin{array}{c} {SNR}_{tc}=\frac{t_{c}}{\sqrt{\sigma_{tc}^{2}}}. \end{array}$$

In the final image, the adipose tissue structures are cancelled out resulting in an almost zero background value. Thus, the SNR of the microcalcification signal in the subtracted image is equal to the CNR [13]. In the rest of this work, we will refer to calculations performed using (5) as the calcification CNR (CNR_*tc*_).

The CNR_*tc*_ has been evaluated for the subtracted digital images of 4 cm thick compressed breast, with composition of 50% glandular and 50% adipose tissue. The calcifications examined were 100 to 500 *μ*m thick in 50 *μ*m increments. Hydroxyapatite (HAp), described chemically as Ca_5_(PO_4_)_3_(OH), was considered as the calcification material [18]. The used detection system consisted of a terbium-doped gadolinium oxysulfide (Gd_2_O_2_S:Tb) phosphor screen (Min-R 2190 with mass thickness of 33.91 mg/cm^2^) coupled to an optical readout device including a CMOS Remote RadEye HR photodiode pixel array [19]. The CMOS photodiode array has a format of 1200 × 1600 pixels, corresponding to an active area of 27 × 36 mm^2^, with a pixel pitch of 22.5 *μ*m [20]. This scintillator was selected due to its high efficiency and imaging properties [21]. The quantum detection efficiency, QDE(*E*_*i*_), and the matching factor, *A*_*s*_, were calculated according to previous studies [20, 21].

The elemental compositions (weight fractions) of the adipose and glandular tissue [22] are shown in Table 1, while for the calcifications and the scintillator materials the corresponding values were calculated by their chemical formula. Based on these data, the mass attenuation coefficients (*μ*/*ρ*) were determined using data published by NISTIR (National Institute of Standards and Technology Interagency Report) [23]. The density values of the above materials are shown in Table 2 [13, 18, 22].

Following mammography regulations, the total skin entrance dose used in the monoenergetic study was 6 mGy [24]. The optimal distribution of the skin entrance dose between the low- and high-energy images was studied by calculating the CNR_*tc*_ in the subtraction image signals as a function of the “low-energy dose ratio,” LDR. The LDR was defined as the ratio of the low-energy dose to the total dose. The optimal dose ratio was determined as the ratio at which CNR_*tc*_ value was maximized.

Since monoenergetic beams cannot be obtained in clinical practice, an approach to monoenergetic beams was employed using polyenergetic X-ray spectra under K-edge filtration [25, 26].

### 2.2. Polyenergetic X-Ray Beams

In the polyenergetic simulation study, the low- and high-energy signals can be calculated by integrating over the energy range in (1) as follows:

(6) $$\begin{array}{c} S_{j}=\overset{E_{max}}{\underset{E_{min}}{\int }}S_{j}\left(\;E_{i}\;\right)dE,\;j=l,h. \end{array}$$

For the implementation of the method using polyenergetic spectra, the mass attenuation coefficients were replaced by effective mass attenuation coefficients in (2) and (3).

Unfiltered spectra were obtained from TASMIP spectral models generated for tungsten (W) anode, for both low energy and high energy [27]. The peak tube voltages were 40 kVp for the low energy (LE), which is the minimum tube voltage available in the radiographic system, and 70 kVp for the high energy (HE).

For the determination of the dose value at which the detector saturates, the detector response curve was measured. Thus, a breast phantom of polymethyl methacrylate (PMMA) and polyethylene (PE) with total thickness of 4 cm [28] was positioned at 62 cm (source to object distance, SOD) from the tube exit of the Del Medical Eureka radiographic system [29] and exposed using tube voltages of 40 kVp and 70 kVp for the low and high energy, respectively. An ionization chamber (Radcal 2026C) was positioned at the surface of the breast phantom. The entrance surface air kerma was measured for a range of tube current-time products. The source to detector distance (SDD) was 66 cm. Both LE and HE images were saved as raw data and 100 × 100 pixels' square regions of interest (ROIs) were measured from each image. The mean pixel value (MPV) and the standard deviation within that region were measured and the relationship between MPV and the detector entrance dose was determined using linear regression. As indicated by the detector response curve of the RadEye HR CMOS sensor, for high-energy spectra, the detector was saturated for doses above 0.42 mGy. For the low energy, the maximum dose value was restricted by the radiographic system performance to 0.37 mGy. Thus, the corresponding unfiltered surface doses that are used in this simulation for the high and low energy were 36.99 and 11.31 mGy, respectively. Additional lower doses were examined by reducing the tube current-time product (mAs).

**Figure 1:**
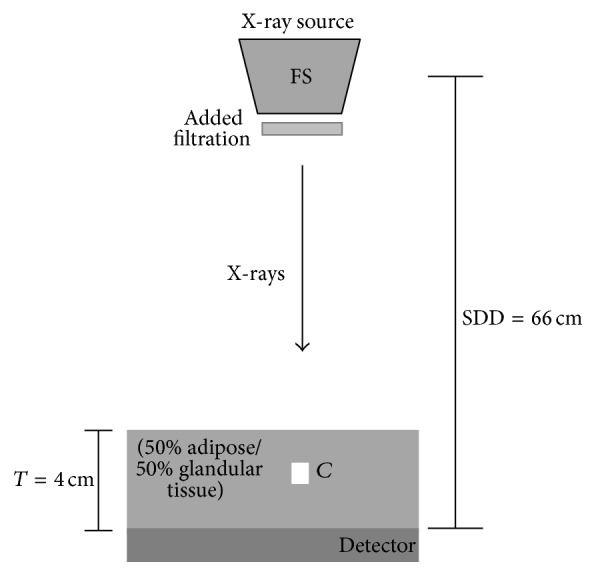
Schematic representation of the simulated set-up.

In order to obtain spectra with mean energies similar to those indicated by the monoenergetic study, various filters based on their K-absorption edge were applied to the beams (K-edge filtering) [26]. In the low-energy beam, three different filter materials were applied: palladium (Pd), silver (Ag), and cadmium (Cd) with K-edges ranging from 24 to 26 keV. For the high-energy beam, five filter materials were applied: copper (Cu), europium (Eu), neodymium (Nd), samarium (Sm), and holmium (Ho). Copper is a low cost filter material commonly used for beam filtration in X-ray systems attenuating low energies in spectra. The other filters used for the HE were rare earth metals with K-edge energies ranging from 43 to 56 keV. The filter thicknesses varied from 10 to 150 *μ*m and 100 to 1500 *μ*m for the low and high energy, respectively. K-edge filtering technique is used, since it produces narrower energy bands of the X-ray spectra leading to an increase of spectral separation [13, 26]. Previous calculations have shown that image noise decreases with greater separation between the low- and high-energy spectra [13]. The breast composition and the detector system used were similar to the monoenergetic study.  Figure 1 shows a schematic representation used for the low and high irradiations of the breast phantom.

The air kerma at the entrance of the breast, after filtration of the X-ray beams, was calculated according to [30]

(7) KamGy=8.77·10−3∑EminEmax1.83·10−6·ΦEi·Ei·μenEiρair,

where Φ(*E*_*i*_) is the filtered X-ray spectrum (photons/mm^2^) at energy *E*. (*μ*_en_(*E*_*i*_)/*ρ*)_air_ is the X-ray mass energy absorption coefficient of air at energy *E* obtained from the literature [23].

Mean glandular dose (MGD) was calculated for various skin entrance doses, considered in this study using [31]

(8) $$\begin{array}{c} MGD=DgN\cdot K_{a}, \end{array}$$

where *DgN* is a numerical factor [32] and *K*_*a*_ is the skin entrance dose defined in (7). MGD was calculated for the low- and the high-energy exposure and then summed to obtain the total mean glandular dose. *DgN* data for a breast thickness of 4 cm, with 0% and 100% glandularity, were obtained from published data [32]. Then, *DgN* values were fitted with a modified Fermi-Dirac distribution function for the 0% and 100% glandular tissues, as follows:

(9) $$\begin{array}{c} f\left(\;x\;\right)=\left(\;\frac{1}{e^{\left(-x+a\right)/b}+1}\;\right)\cdot c-d, \end{array}$$

where *a* = 23.929, *b* = 7.5601, *c* = 1279.2, and *d* = 151 for 0% glandular tissue and *a* = 20.890, *b* = 6.4476, *c* = 1185.6, and *d* = 179 for 100% glandular tissue. For 50% glandular tissue, the mean MGD value was used.

For all filtered spectra, relative Root Mean Square Error (RMSE_rel_) was calculated as an indicative parameter of spectral energy bandwidth. The RMSE_rel_ calculates the divergence between each energy value of the spectrum from the corresponding mean energy [26]. RMSE_rel_ can be expressed as

(10) $$\begin{array}{c} {RMSE}_{rel}=\sqrt{\frac{\sum_{E_{min}}^{E_{max}}\Phi_{\left(E_{i}\right)}{\left(E_{i}-\overset{-}{E}\right)}^{2}}{\sum_{E_{min}}^{E_{max}}\Phi_{\left(E_{i}\right)}\left(E_{i}\right)}}, \end{array}$$

where Φ_(*E*_*i*_)_ are the photons/mm^2^ at each energy, *E*_*i*_ is the energy (keV), and $\overset{-}{E}$ is the mean energy of the spectrum (keV).

## 3. Results and Discussion

### 3.1. Monoenergetic Beams

The optimum pair of beam energies was selected by applying the criterion of CNR_*tc*_ maximization. The difference between the low energy and high energy that maximizes the CNR values was found in the range of 25 to 40 keV. The optimum pair of beam energies was 23 keV and 58 keV for the low and high energy, respectively. Previous calculations with an ideal detector and monoenergetic X-rays predict that 19 keV and 68 keV are the optimal low and high energies for dual energy imaging [10]. Differences in the optimum low-/high-energy pair can be explained on the basis of the detector performance.

**Figure 2:**
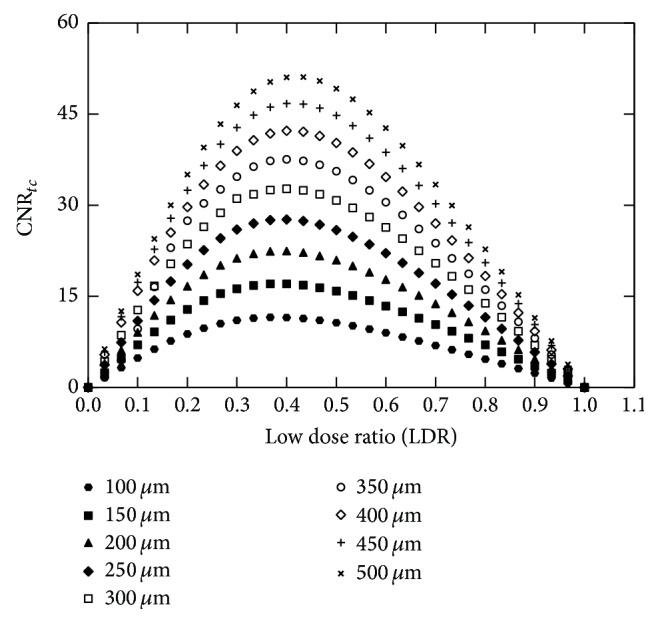
Plots of CNR_*tc*_ as a function of the low dose ratio for all the examined calcification thicknesses.

In Figure 2,  CNR_*tc*_ values are plotted as a function of LDR for all the examined calcification thicknesses. The range of the optimum LDR values, which was defined as the range corresponding to CNR_*tc*_ within the 30% of its maximum, was determined for all the *C* thicknesses considered in the present study. The optimum LDR for 100, 300, and 500 *μ*m calcification thickness ranged from 0.20 to 0.65, 0.20 to 0.66, and 0.20 to 0.66, respectively. Based on the overlap of these ranges, an LDR between 0.20 and 0.65 was considered for all thicknesses. For the minimum examined calcification thickness of 100 *μ*m, a CNR_*tc*_ value of 11.57 can be achieved for the optimum LDR.

### 3.2. Polyenergetic X-Ray Spectra

**Figure 3:**
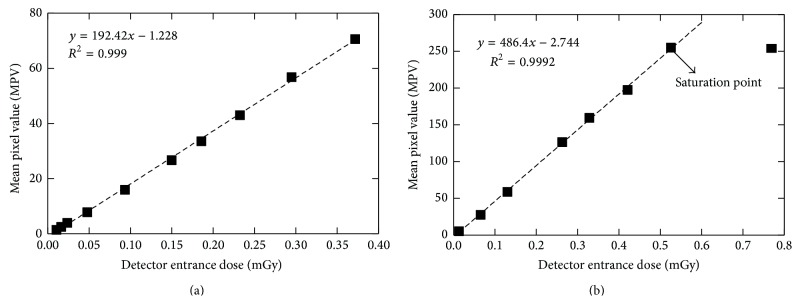
Plot of mean pixel value as a function of the detector entrance dose for the low-energy beam (a) and the high-energy beam (b). The point at the right in (b) was ignored in the linear regression, since it was above the saturation point.

Figures 3(a) and 3(b) show the detector response curves for both the low- and the high-energy beams, respectively. The detector had linear response in the whole exposure range, for the low-energy beam. However, for the high-energy beam, the response of the detector was linear up to 0.42 mGy. Above this value, the detector was saturated. The linear regression correlation coefficient (*R* ^2^) was greater than 0.9990 for the LE beam and 0.9992 for the HE beam.

**Figure 4:**
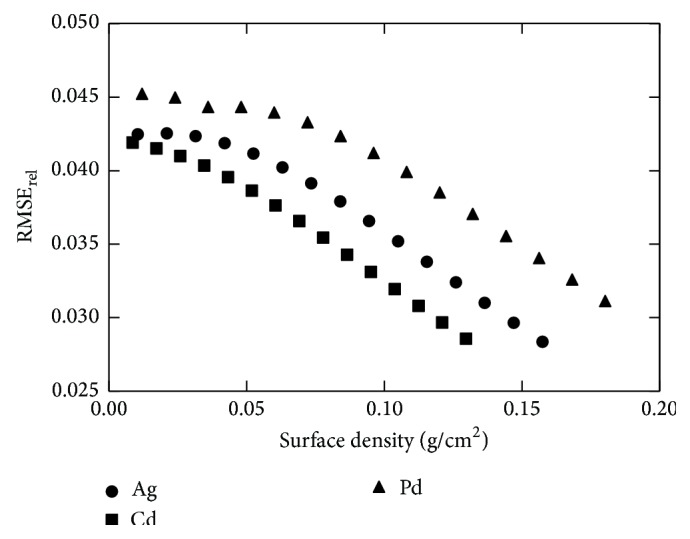
Plots of RMSE_rel_ as a function of surface density (g/cm^2^) for all low-energy filter materials.

Figure 4 shows the RMSE_rel_ curve for all the filter materials examined for the LE, as a function of surface density (g/cm^2^). It is obvious that Cd is the optimum filter material, as it minimizes the RMSE_rel_ over the whole surface density range.

**Figure 5:**
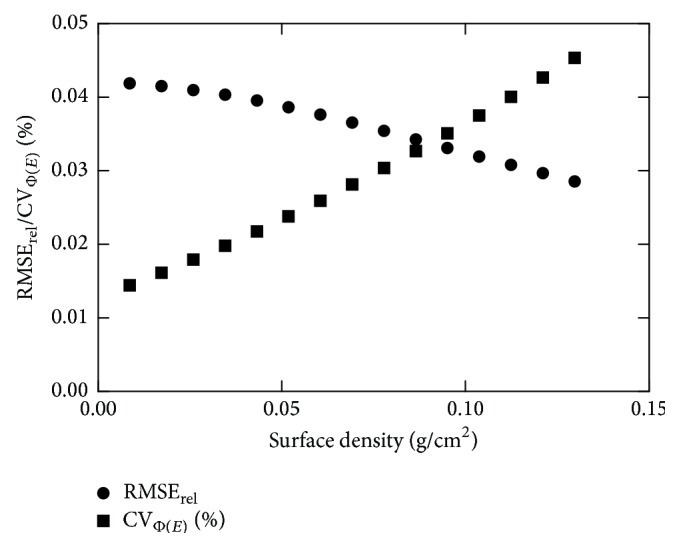
Plots of RMSE_rel_ and CV_Φ(*E*)_ as a function of surface density (g/cm^2^) for Cd.

Figure 5 shows the RMSE_rel_ and the coefficient of variation of the incident number of photons per mm^2^  (Φ(*E*)) at the detector's surface (CV_Φ(*E*)_, where $CV_{\Phi (E)}=\sqrt{\Phi (E)}/\Phi (E)$). The two curves intersect at a point where the RMSE_rel_ is equal to CV_Φ(*E*)_. This point (surface density of 0.086 g/cm^2^) was selected as the optimum filter thickness, corresponding to 100 *μ*m.

**Figure 6:**
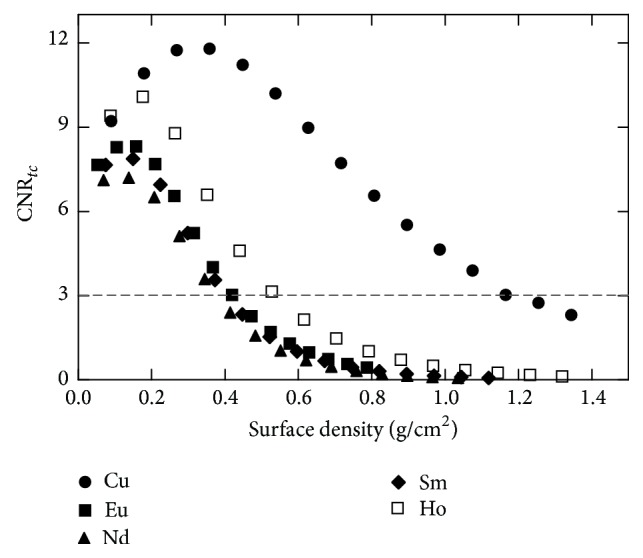
CNR_*tc*_ values as a function of surface density (g/cm^2^) for all high-energy filters, combined with 100 *μ*m Cd (LE filter).

Figure 6 shows the CNR_*tc*_ as a function of surface density (g/cm^2^) for all the HE filters (Cu, Eu, Nd, Sm, and Ho), combined with a Cd filter with a thickness of 100 *μ*m. Copper was selected as the HE filter due to its higher CNR_*tc*_ values compared to the other filter materials.

**Figure 7:**
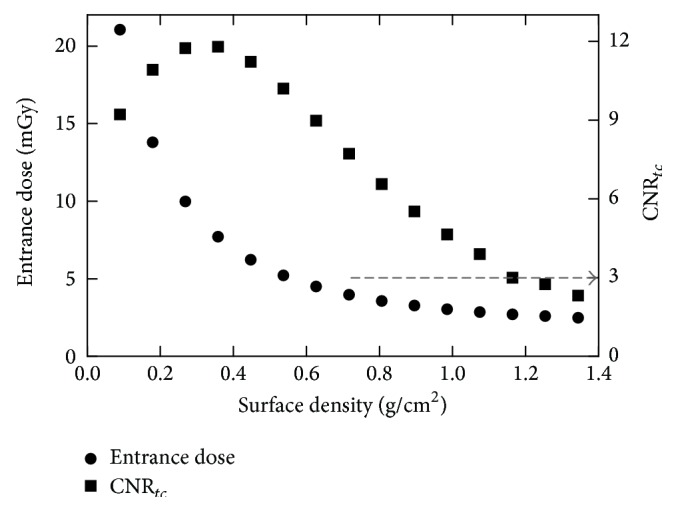
Entrance dose and CNR_*tc*_ values as functions of surface density (g/cm^2^) of Cu (HE filter).

In Figure 7, entrance dose and CNR_*tc*_ are plotted against the surface densities of Cu. For surface densities between 0.896 and 1.075 g/cm^2^, the differences in entrance dose and CNR_*tc*_ were on average 6.63% (7.33%, 5.94%) and 15.95% (15.90%, 16.00%), respectively. Thus, a Cu filter with surface density of 0.896 g/cm^2^ was selected, due to its compromise between entrance dose and CNR_*tc*_. In this thickness, a CNR_*tc*_ of 5.52 (above the threshold, CNR_*tc*_ > 3) can be achieved for a calcification thickness of 150 *μ*m with an entrance dose of 3.52 mGy. When the selected filters of Cd and Cu of 100 *μ*m and 1000 *μ*m were used, the mean energies for the low- and high-energy spectra were 26 keV and 55 keV, respectively.

After filters selection, the CNR_*tc*_ values for all the calcification thicknesses were calculated for different LE and HE entrance surface doses.  Table 3 shows the unfiltered and filtered entrance doses as well as the corresponding LDR values. Entrance dose values and calculated MGD values for 50% glandular tissue are reported in Table 4. Although the screening examination MGD levels for dual energy applications have not yet been established, the MGD levels considered in the present study are those of mammography [33]. In previous dual energy breast imaging studies, the MGD ranged from 1.55 to 2.87 mGy [11, 13, 14]. In this study, the maximum MGD value for 50% glandularity was 2.70 mGy, which is comparable to previous studies and falls within the acceptable levels (MGD < 3 mGy).

**Figure 8:**
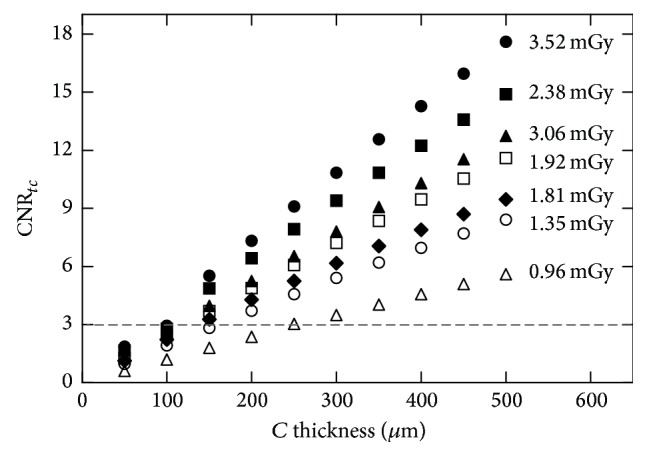
CNR_*tc*_ values as a function of calcification thickness (*μ*m) for various entrance doses.

In Figure 8, CNR_*tc*_ is plotted versus calcification thickness for all total surface doses examined. The higher CNR_*tc*_ values were obtained from the maximum settings (3.52 mGy) for all *C* thicknesses. The minimum detectable *C* thickness was 150 *μ*m, while in previous simulation studies it was 250 *μ*m or higher. When the lower dose was used, the CNR_*tc*_ was found above the threshold when the calcification thickness was 250 *μ*m or higher. Higher doses are expected to have higher CNR_*tc*_ values due to better counting statistics. An inversion between 3.06 and 2.38 mGy curves was observed. This can be attributed to the LDR that affects the calculation of the CNR_*tc*_ (see Figure 2). For the 3.06 mGy, the LDR was 0.75, which is above the optimum LDR range defined to be within the 30% of the maximum CNR_*tc*_ value.

**Figure 9:**
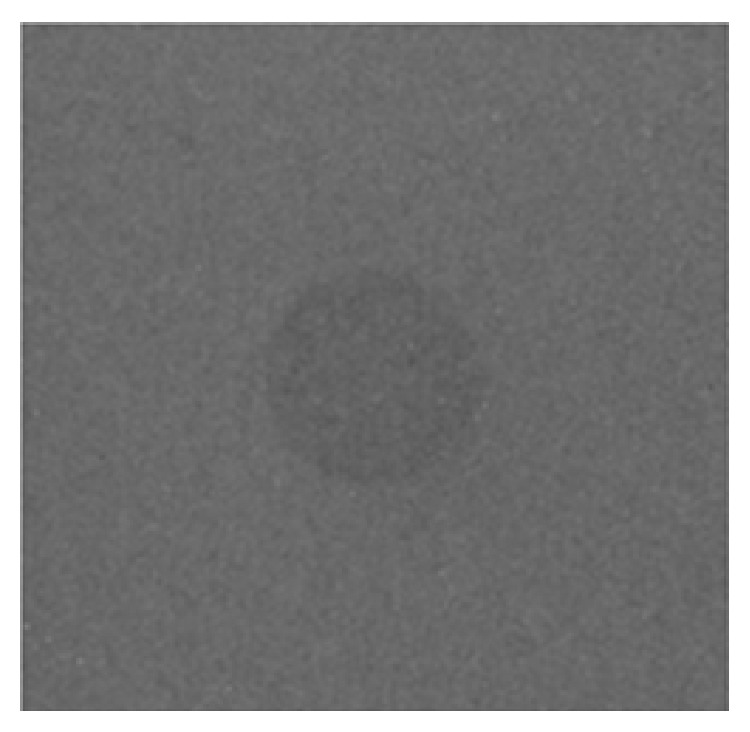
Dual energy subtracted image showing a calcification thickness of 300 *μ*m.

Furthermore, preliminary experimental measurements were carried out using a homogenous phantom.  Figure 9 shows the dual energy subtracted image for a calcification thickness of 300 *μ*m and dose at the entrance of the breast phantom equal to 3.52 mGy. The image was obtained by using 100 *μ*m Cd filter at 40 kVp and 1000 *μ*m Cu filter at 70 kVp. Extended phantom measurements will be held in future studies in order to evaluate the proposed method.

## 4. Conclusions

A simulation study, based on analytical modeling, was performed in order to obtain the optimized irradiation parameters for the detection of the minimum calcification thickness. To this aim, a modified radiographic X-ray unit was considered, in order to overcome the limited kVp range of mammographic units used in previous DE studies, combined with a high resolution CMOS sensor (pixel size of 22.5 *μ*m) for improved resolution. Contrast to noise ratio of the subtracted calcification images was calculated. Various filter materials, based on their K-edge, were examined in order to obtain narrow X-ray spectra. The minimum detectable (CNR > 3) calcification thickness, using monoenergetic beams, was 100 *μ*m, while for polyenergetic X-rays it was 150 *μ*m. The results showed that this method could improve detectability of microcalcifications in breast imaging.

## Figures and Tables

**Table 1:** Elemental composition (weight fractions) of breast phantom materials.

Materials	Elemental composition (weight fraction)			
	H	C	N	O
Adipose tissue	11.2	61.9	1.7	25.1
Glandular tissue	10.2	18.4	3.2	67.7

**Table 2:** Density values for the materials used in the calculations.

Materials	Density (g/cm^3^)
Ca_5_(PO_4_)_3_(OH)	3.18
Gd_2_O_2_S:Tb	7.34
Adipose tissue	0.93
Glandular tissue	1.04

**Table 3:** Unfiltered and filtered entrance surface doses (ESD).

Unfiltered ESD (mGy)			Filtered ESD (mGy)			
LE	HE	Total	LE	HE	Total	LDR
11.31	36.99	48.31	2.28	1.24	3.52	0.64
5.65	36.99	42.64	1.14	1.24	2.38	0.47
2.83	36.99	39.82	0.57	1.24	1.81	0.31
11.31	23.12	34.43	2.28	0.78	3.06	0.75
5.65	23.12	28.77	1.14	0.78	1.92	0.59
2.83	23.12	25.95	0.57	0.78	1.35	0.42
2.83	11.56	14.39	0.57	0.39	0.96	0.59

**Table 4:** Entrance surface dose (ESD) values and MGD for 50% glandularity.

Low energy		High energy	
ESD (mGy)	MGD (mGy)	ESD (mGy)	MGD (mGy)
2.28	1.27	1.24	1.43
1.14	0.62	0.78	1.00
0.57	0.30	0.39	0.50
